# Single-order laser high harmonics in XUV for ultrafast photoelectron spectroscopy of molecular wavepacket dynamics

**DOI:** 10.1063/1.4964775

**Published:** 2016-10-14

**Authors:** Mizuho Fushitani, Akiyoshi Hishikawa

**Affiliations:** 1 Department of Chemistry, Nagoya University, Furo-cho, Chikusa, Nagoya, Aichi 464-8602, Japan; 2 Research Center for Materials Science, Nagoya University, Furo-cho, Chikusa, Nagoya, Aichi 464-8602, Japan

## Abstract

We present applications of extreme ultraviolet (XUV) single-order laser harmonics to gas-phase ultrafast photoelectron spectroscopy. Ultrashort XUV pulses at 80 nm are obtained as the 5th order harmonics of the fundamental laser at 400 nm by using Xe or Kr as the nonlinear medium and separated from other harmonic orders by using an indium foil. The single-order laser harmonics is applied for real-time probing of vibrational wavepacket dynamics of I_2_ molecules in the bound and dissociating low-lying electronic states and electronic-vibrational wavepacket dynamics of highly excited Rydberg N_2_ molecules.

## I. INTRODUCTION

Extreme ultraviolet (XUV) and X-ray pulses from synchrotron radiation and laser induced plasma source have been widely used to study pico- to nano-second processes in a variety of systems in the gas, liquid, and solid phases as well as on surfaces.^1–3^ Recent developments of laser and accelerator technologies have enabled us to study ultrafast phenomena in a shorter time scale. For example, XUV and X-ray free-electron laser^4–7^ can deliver intense femtosecond pulses (∼10 fs) to investigate ultrafast structural dynamics in real time as well as to explore non-linear responses of materials in such high photon energy regions^8–10^ or to prepare exotic targets for photonics.^11^ Laser high-order harmonics generation is a laser-based up-conversion method to obtain coherent ultrashort pulses.^12^ In contrast to the other light sources, laser high-order harmonics can provide a substantially shorter pulse reaching to the attosecond timescale, realizing time-resolved spectroscopy with an unprecedented temporal resolution.^13–16^

High-order harmonics generation occurs in a non-perturbative manner and is explained by the “3-step model”:^17^ (1) electron emission by laser tunneling ionization, (2) acceleration of the freed electron by the laser electric field, and (3) photon emission by the recombination of the freed electron with the ion-core. When a few-cycle pulse is employed as the fundamental, electron trajectory in this recollision process can be controlled by the carrier-envelope phase (CEP) that alters electric-field amplitude under the pulse envelope. It can be chosen so that the burst of high-energy photon emission takes place only once in the few-cycle laser pulse within a duration of ∼100 attosecond. The resultant high-order harmonics is generated as an isolated ultrashort pulse with a broad continuum in the frequency domain.^18^ On the other hand, in a long driving laser pulse (typically >10 fs at 800 nm), the electron recombination occurs every half optical cycle of the fundamental light. The harmonics are generated as a train of pulses in this case, which results in a spectrum with a frequency comb consisting of odd order harmonics.

The cut-off energy of high-order harmonics can reach the soft X-ray or higher energy region, depending on the wavelength and intensity of the fundamental pulse.^12^ Considerable attention has been drawn to laser high-order harmonics especially in the so-called water window region (2–4 nm) in recent years,^19,20^ for time-resolved imaging of biological molecules in solution. Compared to the soft X-ray (0.1–10 nm), larger photon flux can be obtained in the vacuum ultraviolet (VUV, 10–200 nm) and XUV (10–121 nm), where most of the atoms/molecules exhibit large absorption cross-sections by valence or inner-core electron transitions. By applying high-order harmonics as a pump pulse in time-resolved spectroscopy, one can interrogate extremely ultrafast dynamics in highly excited states, such as cascaded Auger processes of Xe,^21^ coherent dynamics of autoionizing states of Xe,^22^ and charge migration in phenylalanine.^23^ Alternatively, harmonics can be used as a probe of ultrafast dynamics triggered by other pump pulses, as demonstrated in transient absorption spectroscopy of valence-shell electron dynamics in Kr^+24^ and two-electron dynamics of He,^25^ and in photoelectron spectroscopy of dissociation dynamics of molecules.^26–29^

Some of these applications favor single-order harmonics. Photoelectron spectroscopy is powerful in studying ultrafast molecular dynamics as electron kinetic energy can directly specify intermediate and/or terminal electronic states involved in the wavepacket motion. When many order harmonics are employed in photoelectron spectroscopy, photoelectron signals (reflecting a dynamical process of interest) can be obscured by spectral overlaps with other photoelectron peaks associated with adjacent harmonic orders. It is therefore preferred to use a single-order harmonic pulse as a probe to prevent spectral congestion. One straightforward approach is to select a particular harmonic order of interest in the frequency domain. There are several approaches proposed for this purpose, using grating pair,^28^ zone-plate,^30^ and dielectric multilayer mirrors,^31–33^ as well as spectral filters.^22,34,35^ The spectral width thus selected determines the shortest pulse duration at the Fourier transformed limit.

For instance, multilayer mirrors coated with SiC/Mg were used for obtaining XUV pulses at 32 nm (*h*ν = 42 eV) generated as the 27th order harmonics of the fundamental at 800 nm.^32^ The reflectance of the SiC/Mg mirror is optimized for the photon energy region of the 27th order harmonics while that for the neighboring order harmonics is suppressed more than an order of magnitude. The 27th order harmonic pulses (∼30 fs) thus obtained were successfully applied to time-resolved photoelectron spectroscopy of unimolecular dissociation of Br_2_ molecules in the *C*^1^Π_1*u*_ state with a temporal resolution of 85 fs.^32^

Alternatively, metal thin foils have been used as band-pass filters for laser high-order harmonics in XUV.^18,22,24,34,35^ Although optical properties such as transmission photon energies and bandwidths are determined by materials of thin foils, they offer a simple and robust way for the single harmonics order selection. In this contribution, we describe our recent work on the single-order harmonics generation in XUV by using an indium foil^35^ and its applications to ultrafast photoelectron spectroscopy.

## II. EXPERIMENTAL

**FIG. 1.**
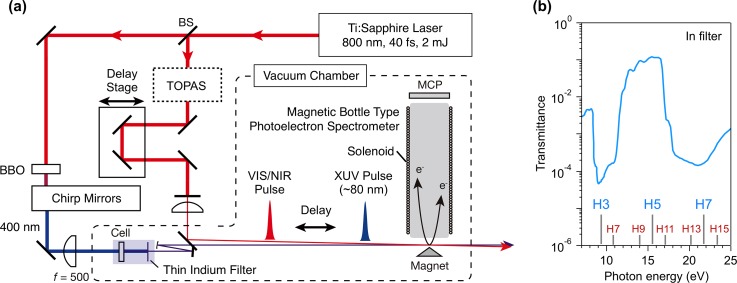
(a) Schematic diagram of the experimental setup for time-resolved photoelectron spectroscopy with single-order harmonics at 80 nm. (b) Transmittance of an indium filter (0.1 μm thick). Energy positions of the high order harmonics (H3–H7 for 400 nm and H7–H15 for 800 nm) are indicated by bars.

Figure 1(a) shows a schematic diagram of our experimental setup for ultrafast photoelectron spectroscopy. The output of the Ti:Sapphire laser system (800 nm, <40 fs, 2 mJ/pulse) was divided into two parts. One was frequency-doubled by a BBO crystal and focused by a plano-convex lens (*f* = 500 mm) to a cylindrical cell containing rare gas (Kr, Xe) in a high vacuum chamber. Generated high-order harmonic pulses were transmitted through a thin metal filter and focused by a concave mirror (*f* = 1000 mm) to a gaseous target in the interaction region of a magnetic bottle spectrometer. The other laser output in near-infrared (NIR) was used to generate ultrashort visible (VIS) pulses by an optical parametric amplifier (TOPAS-C, Light Conversion Ltd.) to pump iodine molecules or directly used as a probe for the Rydberg wavepackets of N_2_ without wavelength conversion. The VIS/NIR laser beam was introduced into the spectrometer with a small angle (0.25°) to the high-order harmonics. Electrons from the target molecules were guided to a micro-channel plate (MCP) detector by an inhomogeneous magnetic field of a cone-shape permanent magnet as well as a homogeneous magnetic field from a solenoid. Electron signals were counted by using an amplifier-and-timing discriminator (9327, Ortec) and a time-to-digital converter (TDC8, RoentDek). A typical resolution (Δ*E*_kin_) at an electron energy (*E*_kin_) was *E*_kin_/Δ*E*_kin_ ∼ 50 with a 1.5 m-long time-of-flight tube.

To select single-order harmonics from the nonlinear media, we employed an indium filter (0.1 *μ*m thickness, Ni-mesh support, Lebow Co.) having transmittance in the XUV region of *hν* = 13–16 eV (Fig. 1(b)). In our approach, laser pulses at 400 nm, instead of 800 nm, are employed as the driving laser. Since the 5th order harmonics at 80 nm (15.5 eV) falls within the transmittance band of an indium filter, it can be selected from other harmonics. It should be noted that when an 800 nm pulse is used, both the 9th and 11th order harmonics are transmitted (see Fig. 1(b)). Instead, for the 400 nm pulse, care should be taken to pre-compensate the spectral dispersion by chirp mirrors, because dispersion introduced by optical windows, lens, and air during the propagation is more significant in the UV range than in NIR. To demonstrate the single-order harmonics selection by using an indium filter, photoelectron spectroscopy of iodine molecules was carried out.^35^ Without indium filters, photoelectron peaks associated with the single 5th and 7th order harmonics as well as the combination of the 3rd and 5th order harmonics with the fundamental were identified in the spectrum. On the other hand, by inserting an indium filter, these peaks disappeared except for the peaks corresponding to the 5th order harmonics.^35^

## III. APPLICATION TO ULTRAFAST PHOTOELECTRON SPECTROSCOPY

### A. Vibrational wavepacket dynamics of I_2_

Molecular iodine in the low-lying electronic states has been subjected to a variety of time-resolved studies based on light-induced fluorescence,^36–38^ four-wave mixing,^39,40^ ion mass spectrometry,^41,42^ and zero-kinetic energy (ZEKE) photoelectron spectroscopy.^43^ In addition to periodic vibrational motions, coherent phenomena such as fractional revivals^43^ and quantum ripples due to wavepacket interference^36^ have been identified in the BΠ30+u state. In most experimental observations, however, wavepacket motion is probed in a confined region around turning points where optical transition dominantly takes place because of the Franck-Condon principle. This limitation could be removed when wavepacket is projected onto (dissociative) ionic state where spatial information on wavepacket motion can be reflected to a change of photoelectron or ion kinetic energy. Such measurements with ultrashort pulses are of significant importance to identify how molecular coherence collapses spatiotemporally during the wavepacket evolution. Here, we perform ultrafast photoelectron spectroscopy of I_2_ molecules as a prototype system by using single-order harmonics in XUV in order to demonstrate real-time probing of the wavepacket dynamics over a wide range of the internuclear coordinate.

**FIG. 2.**
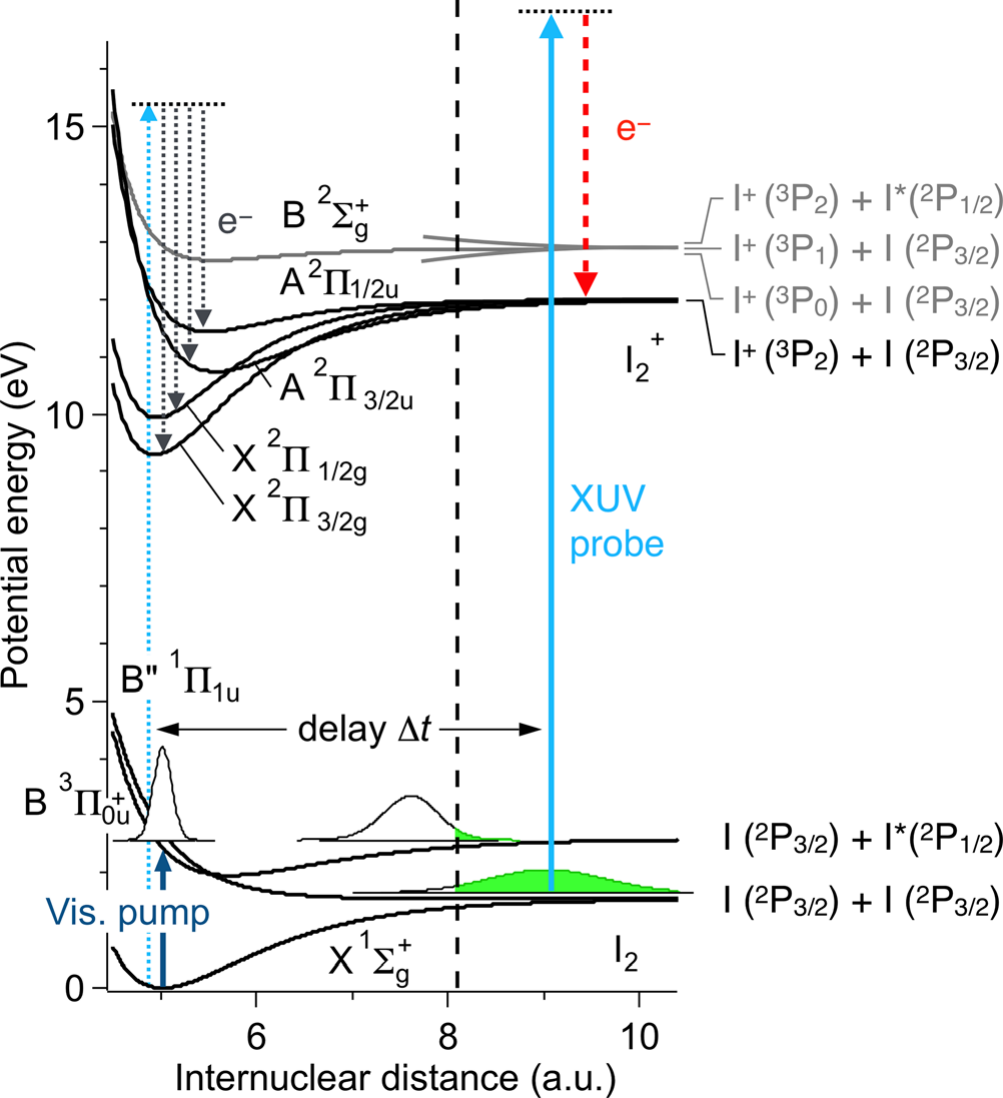
Potential energy curves represent relevant electronic states of I_2_ and I_2_^+^.^45^ Dynamics of vibrational wavepackets launched on excited I_2_ molecules by a visible pump pulse is probed by an XUV ultrashort laser pulse introduced with a time delay Δ*t*. The nuclear wavepackets at Δ*t* = 0 and 120 fs are shown for the pump wavelength of 490 nm. The density of wavepacket at internuclear distances larger than 8.1 a.u. is taken as the atomic signal (see text).

Figure 2 shows relevant potential energy curves of I_2_ and I_2_^+^ molecules.^44,45^ An ultrashort pump laser pulse (490 nm, *hν* = 2.53 eV, ∼90 fs) launches nuclear wavepackets in both the bound BΠ30+u and the repulsive B″Π11u states. These vibrational wavepackets propagate independently on the BΠ30+u and the B″Π11u potential curves. Since the pump photon energy is above the dissociation threshold of the bound BΠ30+u, both of these wavepackets eventually dissociate as I_2_(BΠ30+u) → I(^2^P_3/2_) + I*(^2^P_1/2_) and I_2_(B″Π11u) → I(^2^P_3/2_) + I(^2^P_3/2_), respectively.

**FIG. 3.**
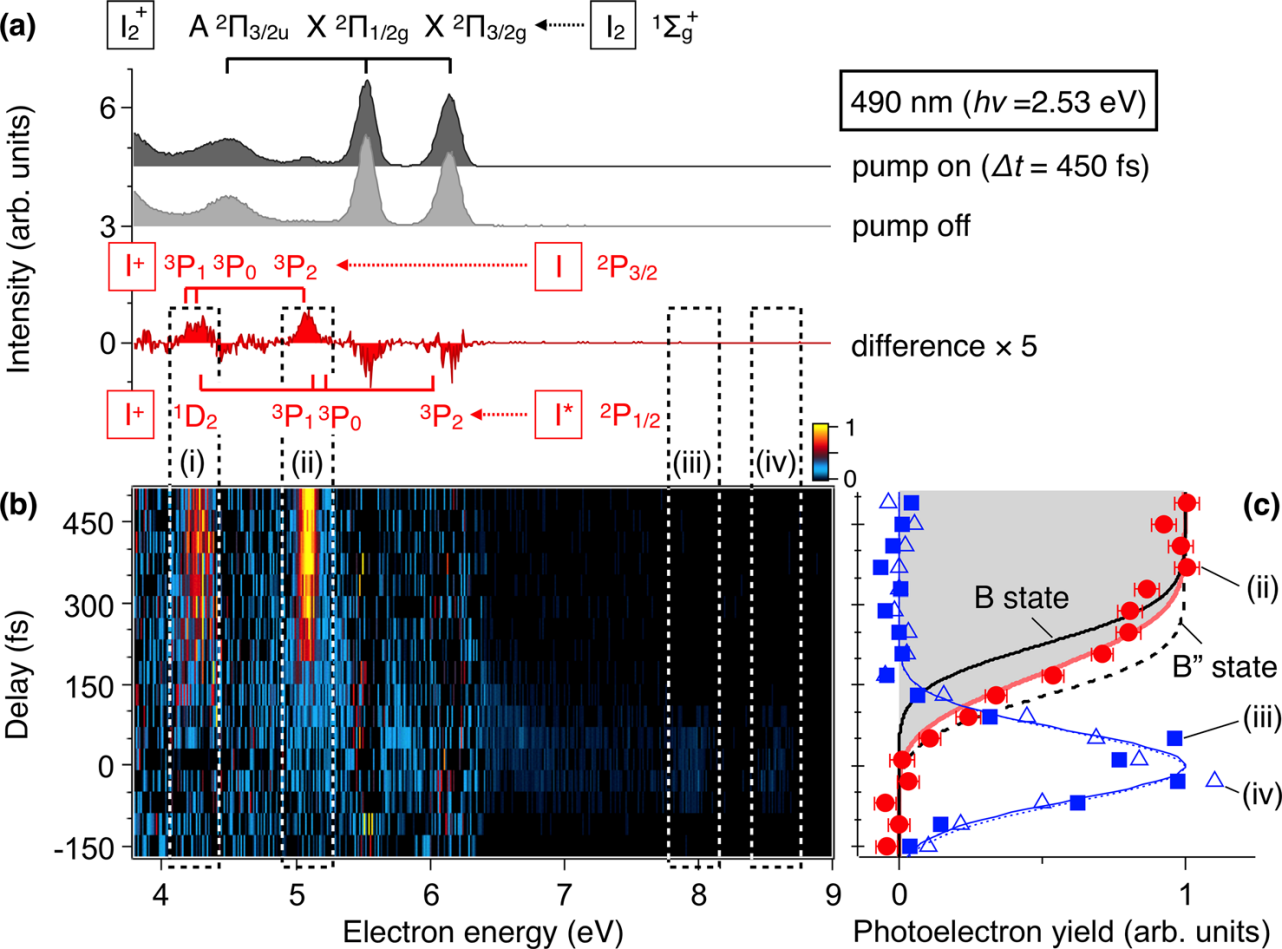
(a) Photoelectron spectra of I_2_ recorded with the pump pulse at 490 nm and the probe pulse at 80 nm with a delay of Δ*t* = 450 fs. Photoelectron spectra obtained without the pump pulse and the difference between pump-on and pump-off spectra are also shown. (b) Time evolution of the difference photoelectron spectrum as a function of Δ*t*. (c) Photoelectron intensities plotted against the time delay Δ*t* for peak (ii) (solid circles), peak (iii) (solid squares), and peak (iv) (open triangles). Theoretical atomic signals obtained by wavepacket simulation propagating on the BΠ30+u state (solid, black), the B″Π11u state (dashed, black), and both states (solid, red).

The wavepacket dynamics evolving on these two different potential curves is probed by the ultrashort XUV pulses (80 nm, 15.5 eV, 121 fs) introduced with a time delay (*Δt*). Figure 3(a) shows the photoelectron spectrum of I_2_. Since the XUV photon energy exceeds the ionization threshold (9.3 eV) of the I_2_ molecule, photoelectrons from the ground state ($X^{1}\Sigma_{g}^{+}$) to $X^{2}\Pi_{1/2g}$ and $X^{2}\Pi_{3/2g}$ states of I_2_^+^ are observed at 5.5 eV and 6.2 eV in the spectrum, respectively. When the pump pulse is introduced at *Δt* = 450 fs, new peaks (i) and (ii) appear at 4.3 eV and 5.1 eV. From the energy conservation, these peaks can be assigned to photoelectrons from iodine atoms in the ground (^2^P_3/2_) and the excited (^2^P_1/2_) states.

The evolution of the difference photoelectron spectrum is shown in Fig. 3(b). Both the peak (i) and the peak (ii) show a similar behavior exhibiting a rapid increase after the irradiation of the pump pulse at *Δt* = 0. The integrated intensity of peak (ii) is plotted as a function of *Δt* in Fig. 3(c), showing that the signal presents a monotonic increase to reach a plateau at *Δt* ∼ 300 fs. To understand how the dissociation dynamics in the I_2_ excited states is reflected in the observed photoelectron spectra, vibrational wavepacket simulation was carried out separately for the BΠ30+u and B″Π11u states by using the split-operator method.^46^ The wavepacket launched on the B″Π11u potential curve immediately starts to leave the Franck-Condon region of the I_2_ ground state to reach the dissociation limits I(^2^P_3/2_) + I(^2^P_3/2_) in ∼200 fs (Fig. 2). The wavepacket in BΠ30+u shows slightly slower dissociation dynamics (∼300 fs) to I(^2^P_3/2_) + I*(^2^P_1/2_) due to the bound character of the excited state.

To compare with the experimental results of the peak (ii), the atomic photoelectron signals are assumed proportional to the wavepacket density ${\left|\psi (R,t)\right|}^{2}$ integrated over the internuclear distance *R* ≥ 8.1 a.u., where the photoelectron energy falls within the observed width (0.24 eV) of the peak at 5.1 eV. The obtained results in Fig. 3(c) show an earlier increase for the B″Π11u state (dashed, black) than BΠ30+u (solid, black) as expected. It should be noted that the experimental results cannot be explained by the contributions from the BΠ30+u state or that from the B″Π11u state alone, because both curves have steeper slopes than the experimental data. Therefore, to reproduce the experimental results, the contributions from these two states should be added with appropriate weights. In our previous study, the peak at 5.1 eV is assigned to photoionization from the atomic ground state, I(^2^P_3/2_) → I^+^(^3^P_2_) + e^–^.^35^ The contribution from the excited fragment, I*(^2^P_1/2_) → I^+^(^3^P_1_) + e^–^, is considered negligible because the corresponding peak to another spin-orbit state, I*(^2^P_1/2_) → I^+^(^3^P_0_) + e^–^, is missing in the spectra. In such a case, a weight ratio of the two curves can be obtained from the absorption cross-sections (1.1 Mb and 0.65 Mb) of the BΠ30+u and B″Π11u states from the ground state^47^ with the latter multiplied by 2 to account for the yields of the I(^2^P_3/2_) atom. The curve simulated with relative weights of 0.46 and 0.54 thus obtained is plotted in Fig. 3(c), showing a good agreement with the observed data. These results clearly demonstrate that ultrafast molecular dissociation evolving in the two different electronic states is simultaneously monitored in real time by photoelectron spectroscopy with ultrashort XUV pulses.^35^

The difference photoelectron spectra in Fig. 3(b) show additional weak peaks (iii) and (iv) at 7.9 eV and 8.6 eV at *Δt* = 0 fs, which disappear in a short time scale (∼100 fs) as shown in Fig. 3(c). Since the difference between photoelectron energies observed at *Δt* = 0 fs and the original photoelectron peaks at 5.5 eV and 6.2 eV (to the ground states of I_2_^+^) agrees with the pump photon energy (*hν* = 2.53 eV), these weak peaks can be assigned to photoelectrons from the excited states of I_2_. Figure 3(b) shows that the peak (iii) at 7.9 eV is accompanied by a tail-like feature extending to *Δt* ∼ 150 fs, which can be attributed to the evolution of the vibrational wavepacket in the excited states.

**FIG. 4.**
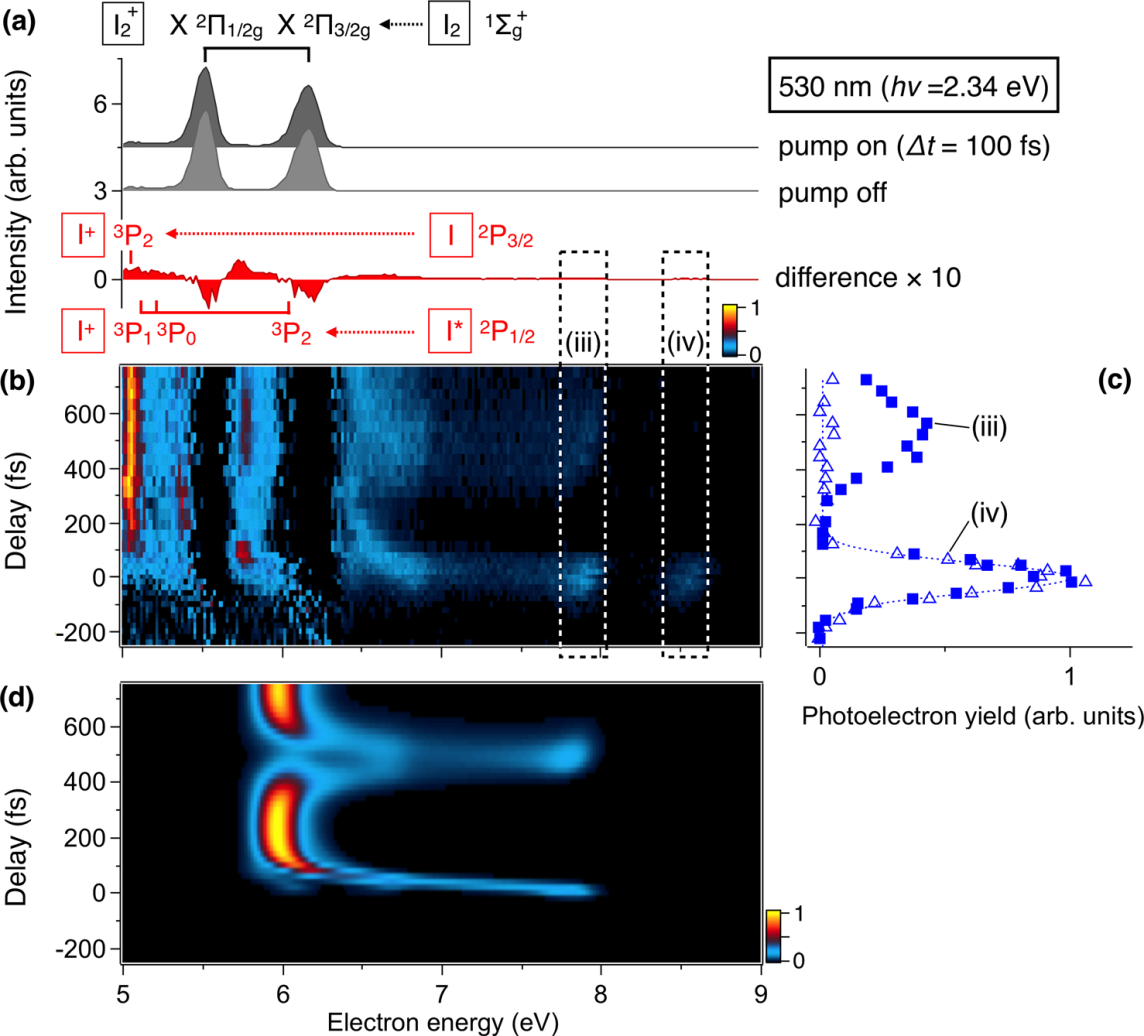
(a) Photoelectron spectra of I_2_ by using a pump pulse at 530 nm and a probe pulse at 80 nm with a delay of Δ*t* = 100 fs. Photoelectron spectra obtained without the pump pulse and the difference between pump-on and pump-off spectra are also shown. (b) Time evolution of the difference photoelectron spectrum as a function of Δ*t*. (c) Photoelectron intensities plotted against the time delay Δ*t* for peaks (iii) and (iv). (d) Evolution of photoelectron spectra simulated incorporating the contributions from the XΠ21/2g, AΠ23/2u, and AΠ21/2u final states of I_2_^+^ (see text for details).

To illustrate how the propagation of a vibrational wavepacket is mapped to the XUV photoelectron spectrum, we reduced the pump photon energy to 2.34 eV (530 nm) to excite bound vibrational levels in the BΠ30+u state. At this photon energy, a vibrational wavepacket is prepared by a coherent superposition of the vibrational levels (*v* ∼ 30). The obtained results are shown in Fig. 4. The bound BΠ30+u state dominates the photoabsoption from the ground state with a large absorption cross-section σ of 2.71 Mb,^47^ but contributions from the repulsive B″Π11u state (σ = 0.41 Mb (Ref. 47)) are also visible as the appearance of the atomic peak (ii) at 5.1 eV at a large time delay.

The difference photoelectron spectra in Fig. 4(b) show the appearance of the peak (iii) and the peak (iv) at 7.8 and 8.5 eV around *Δt* = 0 fs. The evolution of these peak intensities is plotted in Fig. 4(c), showing that the latter appears only around *Δt* = 0 fs. The peak (iv) is therefore attributed to a sideband of the $X^{2}\Pi_{\mathrm{3/2}g}$ photoelectron peak, which appears only when the visible and XUV laser pulses are overlapped in time. The same applies to the peak (iii), assigned to the sideband of the $X^{2}\Pi_{\mathrm{1/2}g}$ peak. However, the latter contains an additional component, which recurs at a longer time delay at *Δt* ∼ 500 fs. From the energy conservation, this component can be attributed to the photoelectrons from the BΠ30+u state to the I_2_^+^ $X^{2}\Pi_{\mathrm{1/2}g}$ state. Indeed, the recurrence period is in good agreement with the classical vibrational period (440 fs) for *v* = 30 in the BΠ30+u state. The difference between the XΠ23/2g and XΠ21/2g components is explained as follows. The main electronic configurations are σ_*g*_^2^π_*u*_^4^π_*g*_^3^σ_*u*_^1^ for the BΠ30+u state and σ_*g*_^2^π_*u*_^4^π_*g*_^3^σ_*u*_^0^ for the XΠ23/2g and XΠ21/2g states, respectively. Since the removal of the σ_*u*_ electron in the BΠ30+u state can only change the *Ω* value from 0^+^ to 1/2, the photoionization from the BΠ30+u state favors the XΠ21/2g state rather than the XΠ23/2g state.

**FIG. 5.**
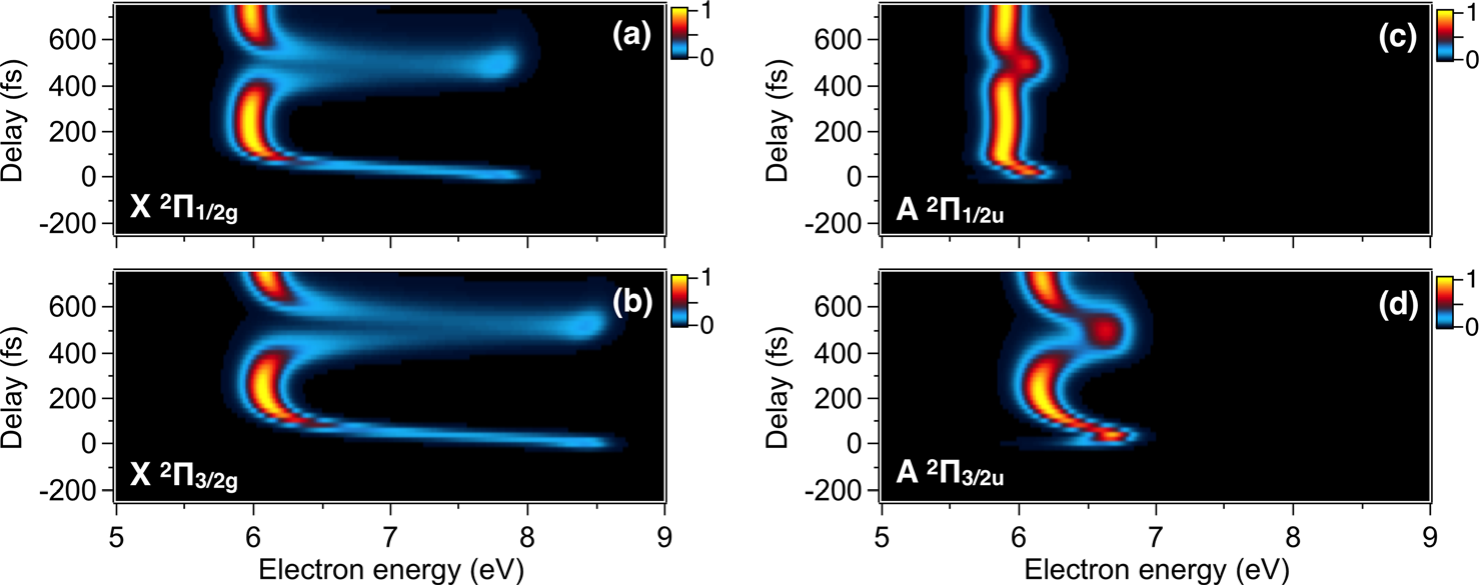
Time-resolved photoelectron spectra calculated for the vibrational wavepacket in the BΠ30+u state with different final states in I_2_^+^: (a) XΠ21/2g, (b) XΠ23/2g, (c) AΠ21/2u, and (d) AΠ23/2u. The pump and probe photon energies are 2.53 eV (530 nm) and 15.5 eV, respectively (see text for details).

To understand the observed vibrational dynamics, wavepacket simulation for the BΠ30+u state was carried out. The time-resolved photoelectron spectra were separately calculated for each final state (Fig. 5) by the time-dependent perturbation theory.^48^ Figure 4(c) shows the simulated photoelectron spectra obtained as a weighted sum of the relevant final states, XΠ21/2g, AΠ23/2u and AΠ21/2u. The contributions from the AΠ23/2u and AΠ21/2u states are adjusted to reproduce the experimental results and set to 1/10 of the XΠ21/2g states, respectively. The small weights are attributed to the fact that the transition of two electrons is required for photoionization from the BΠ30+u state (σ_*g*_^2^π_*u*_^4^π_*g*_^3^σ_*u*_^1^) to the AΠ2u states (σ_*g*_^2^π_*u*_^3^π_*g*_^4^σ_*u*_^0^). The simulated result shows a periodic behavior in the photoelectron energy range of 6–8 eV, due to the wavepacket oscillations in the BΠ30+u state. The signals at 6.0 and 7.9 eV correspond to wavepacket at the outer and inner turning points of the BΠ30+u state, respectively.

It should be noted that there exist a number of excited states of I_2_^+^ converging to the I^+^(^3^P_2_) + I*(^2^P_1/2_), I^+^(^3^P_0_) + I(^2^P_3/2_), and I^+^(^3^P_1_) + I(^2^P_3/2_) asymptotes, located 0.943 eV, 0.799 eV, and 0.879 eV above the lowest dissociation limit.^44^ This implies that contributions from these final states appear at photoelectron energies about ∼1 eV lower than that for the XΠ2g and AΠ2u final states for the vibrational wavepacket probed near the outer turning points. Indeed, remnants of oscillatory structures are seen in Fig. 4(b) in the photoelectron energy range (5–6 eV) where the contributions from the excited final states are expected.

The present results demonstrate that the single-order harmonics at 80 nm is a powerful probe for time-resolved photoelectron spectroscopy to monitor nuclear wavepacket motion. The high photon energy allows us to trace the dynamics of target molecules all the way from the initial stages of the photo-induced processes to the final products. On the other hand, the photoelectron signals from the target molecules in the ground state can be an obstacle, as it masks the time-dependent components in the photoelectron spectra. In the present case, the vibrational dynamics corresponding to the photoelectron energy regions around 5.5 and 6.2 eV are not clearly visible due to the spectral overlap with the strong XΠ21/2g and XΠ23/2g peaks (see Figs. 3(b) and 4(b)). Further developments of tunable ultrashort XUV light sources will be necessary in this respect.

### B. Rydberg wavepacket dynamics of N_2_

Single-order harmonics can be used as a pump to study ultrafast dynamics in highly excited states. Rydberg wavepackets, formed by coherent superposition of highly excited Rydberg states, exhibit dynamics on a variety of time scales depending on the (effective) principle quantum number (*n*).^49^ Since the classical orbiting period of a Rydberg electron scales with *n*^3^,^50^ the time scale of electron dynamics rapidly increases as *n* increases and reaches the femto- to pico-second range at *n* ∼ 10, which is comparable with those of vibrational and rotational degrees of freedom in a molecule. Therefore, unlike atomic systems,^51^ molecular Rydberg wavepacket exhibits more complex dynamics due to the interplay between electron and nuclear degrees of freedom.

Rydberg states of molecular nitrogen have been subjected to a number of detailed studies by absorption,^52–54^ ZEKE^55^ and fluorescence spectroscopy,^56^ collision experiments,^57^ and also by *ab initio* calculations.^58–60^ Picosecond lifetime measurements were reported for the Rydberg states in the 95–96 nm range (13 eV), $c_{4}\prime {\;}^{1}\Sigma_{u}^{+}$ (*v* = 0–2),^61^ $b^{1}\Pi_{u}$(*v* = 1).^62^ Recently, high-order laser harmonics was applied to study transient Fano resonances on autoionizing $B^{2}\Sigma_{u}^{+}$ 3 d*π*_*g*_ and 4“*s”σ*_*g*_ states located above the ionization threshold (15.58 eV).^63^ In the present study, time-resolved photoelectron spectroscopy with single-order harmonics at 80 nm is carried out to investigate ultrafast coherent dynamics of Rydberg states which are converging to the $X^{2}\Sigma_{g}^{+}$ as well as $A^{2}\Pi_{u}$ states of the N_2_^+^ ion.

**FIG. 6.**
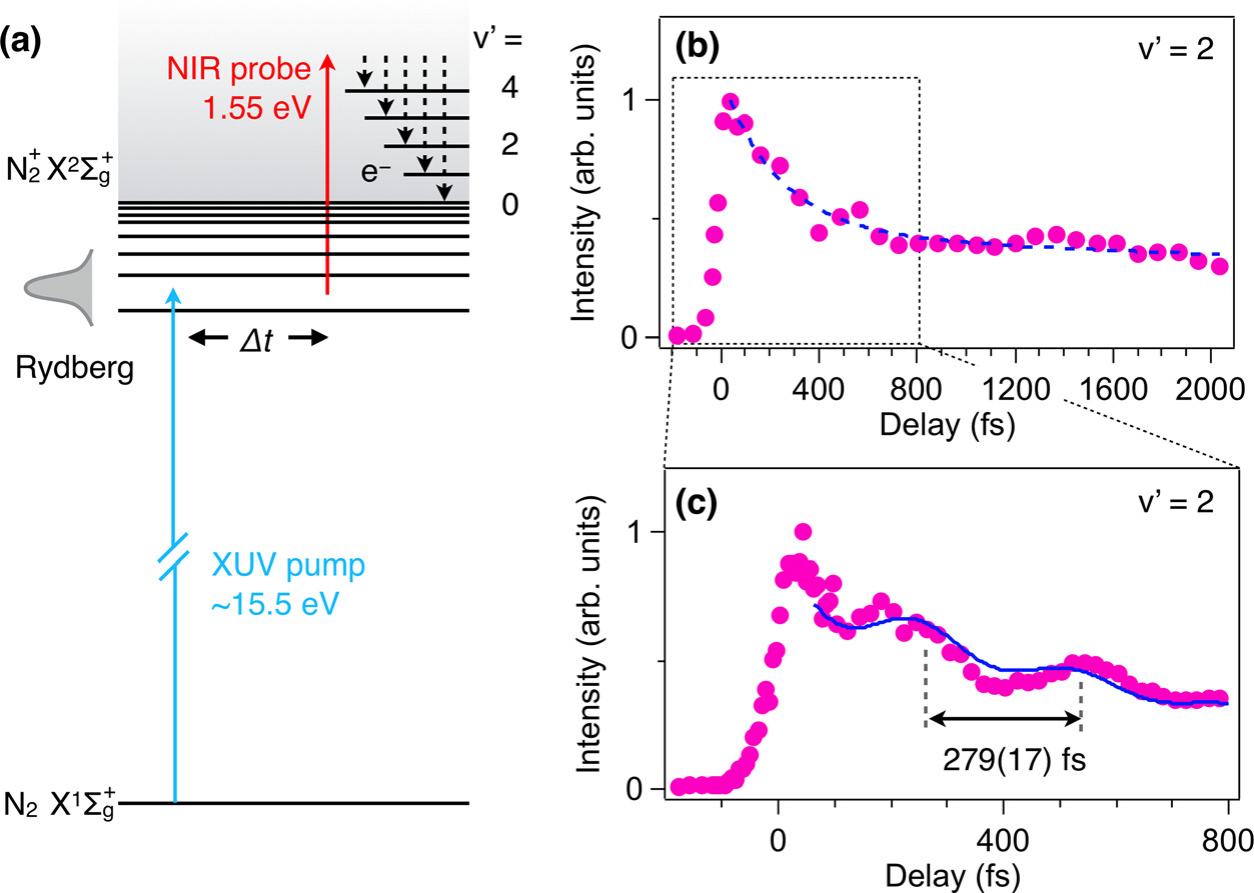
(a) Schematics of XUV-pump and NIR-probe photoelectron spectroscopy of N_2_ Rydberg wavepacket dynamics. (b) Integrated intensities of photoelectron signal corresponding to the *v*′ = 2 peak as a function of pump-probe time delay up to 2.04 ps. The signal decrease is characterized by double exponential decay with 290(40) fs and 9(7) ps. (c) The same as (b) but in the range of −176 to 790 fs with a finer scan step.

The single-order harmonics at 80.4 nm (15.42 eV) covers several absorption peaks of N_2_ in the bandwidth (∼0.10 eV) to create a wavepacket consisting of *n*pπ(0) (*n* = 9–13), 9pσ(0), 10pσ(0), 6pπ(1), 5pπ(2), 5pσ(2), 4pπ(4), 8f(0), and 9f(0) Rydberg states converging to $X^{2}\Sigma_{g}^{+}$ and 3dδ(1), 3dσ(2) and 4sσ(1) to $A^{2}\Pi_{u}$,^54^ where numbers in the parentheses represent the vibrational quantum numbers. Time evolution of the Rydberg wavepacket is probed by a time-delayed ultrashort NIR pulse (800 nm) ionizing to the N_2_^+^ $X^{2}\Sigma_{g}^{+}$ state (see Fig. 6(a)).

The recorded photoelectron spectrum exhibits five peaks corresponding to the *v*′ = 0–4 final vibrational levels of N_2_^+^$X^{2}\Sigma_{g}^{+}$. Compared with the conventional photoelectron spectrum by using the He II light source,^64^ three extra peaks to the *v* ′ = 2–4 levels are identified. The time evolution of the integrated intensity of the *v*′ = 2 peak is plotted in Fig. 6(b) as a function of the pump-probe time delay up to ∼2 ps. The signal shows a steep rise around 0 fs and exhibits a rapid decay characterized by a double exponential function with *τ*_1_ = 290 (40) fs and *τ*_2_ = 9(7) ps lifetime components.

The temporal profile of the *v*′ = 2 peak recorded with a finer step is shown in Fig. 6(c), which exhibits a clear modulation in intensity with a period of 279(17) fs up to Δ*t* = 800 fs. The oscillation period can be attributed to the coherent dynamics between Rydberg states pumped by the XUV laser pulse at 80.4 nm. In this wavelength region, there are two dominant absorption bands around 80.6 and 80.1 nm.^54^ The former exhibits a substructure with two main peaks, one consisting of 6pπ(1), 8f(0), and 3dδ(1) and the other consisting of 5pπ(2), 9pπ(0), and 4pπ(4). The energy difference between these two peaks is about 110 cm^−1^, which corresponds to a period of 300 fs in good agreement with the observed period. Since these bands contain Rydberg states with different principal quantum numbers, conversing to different electronic and vibrational states of N_2_^+^, the observed coherent dynamics would be understood in terms of a superposition of wavepackets of (i) Rydberg electron motion, (ii) electron motion in the ion core, $X^{2}\Sigma_{g}^{+}$ and $A^{2}\Pi_{u}$, and (iii) vibrations of the ion core. Such a Rydberg system exhibiting interplay between different degrees of freedom should be interesting targets for coherent control of molecular dynamics as demonstrated with NO.^65^

## IV. SUMMARY

We presented a simple and robust approach for single-order harmonic generation in XUV, by utilizing 400-nm fundamental laser pulses and an indium thin foil filter. The applications to ultrafast photoelectron spectroscopy on (1) vibrational wavepackets of bound and repulsive excited states of I_2_ and (2) electronic-vibrational Rydberg wavepackets of N_2_ are demonstrated, showing that the unique approach generating XUV single-order laser harmonics is promising for studying ultrafast molecular wavepacket dynamics.
